# Supplementary figures and images for: The Arabidopsis V-ATPase is localized to the TGN/EE via a seed plant-specific motif
Source: eLife. 2020 Nov 25;9:e60568. doi: 10.7554/eLife.60568 (PMC7717909; doi:10.7554/eLife.60568)

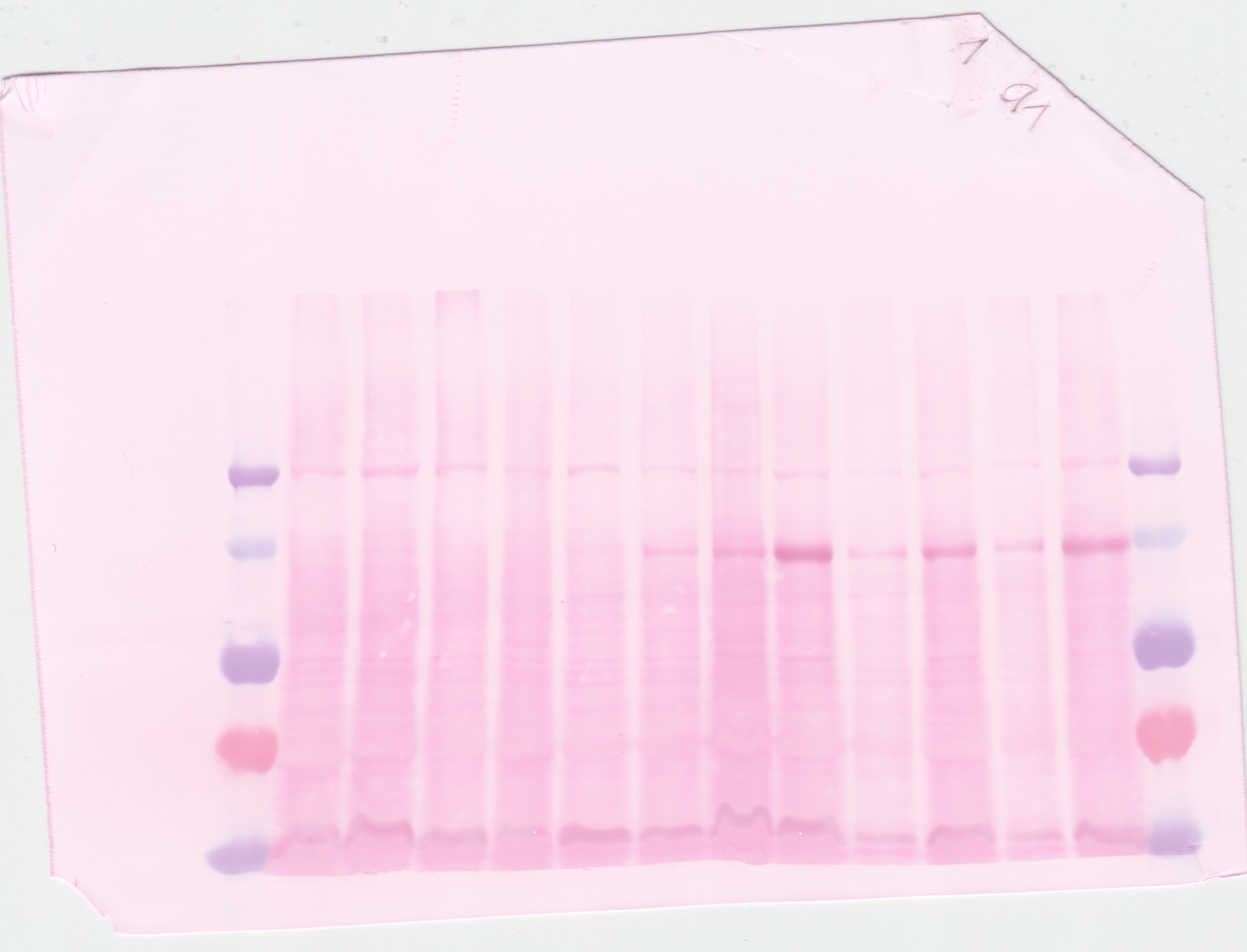

Supplement: Figure 7—figure supplement 2—source data 1. [file elife-60568-fig7-figsupp2-data1.zip › 1a1 001.tif]

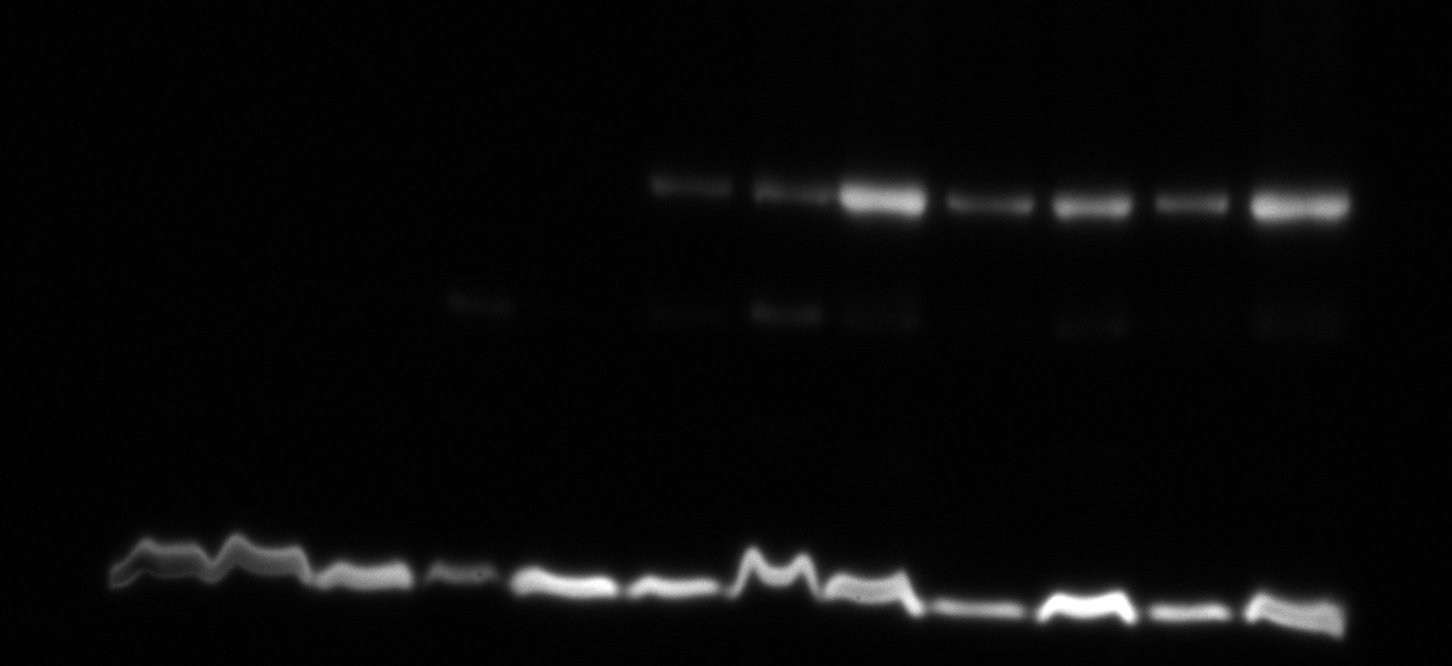

Supplement: Figure 7—figure supplement 2—source data 1. [file elife-60568-fig7-figsupp2-data1.zip › 1_mutations _anti VHA_B 2 sec,1-1. scan_raw.tif]

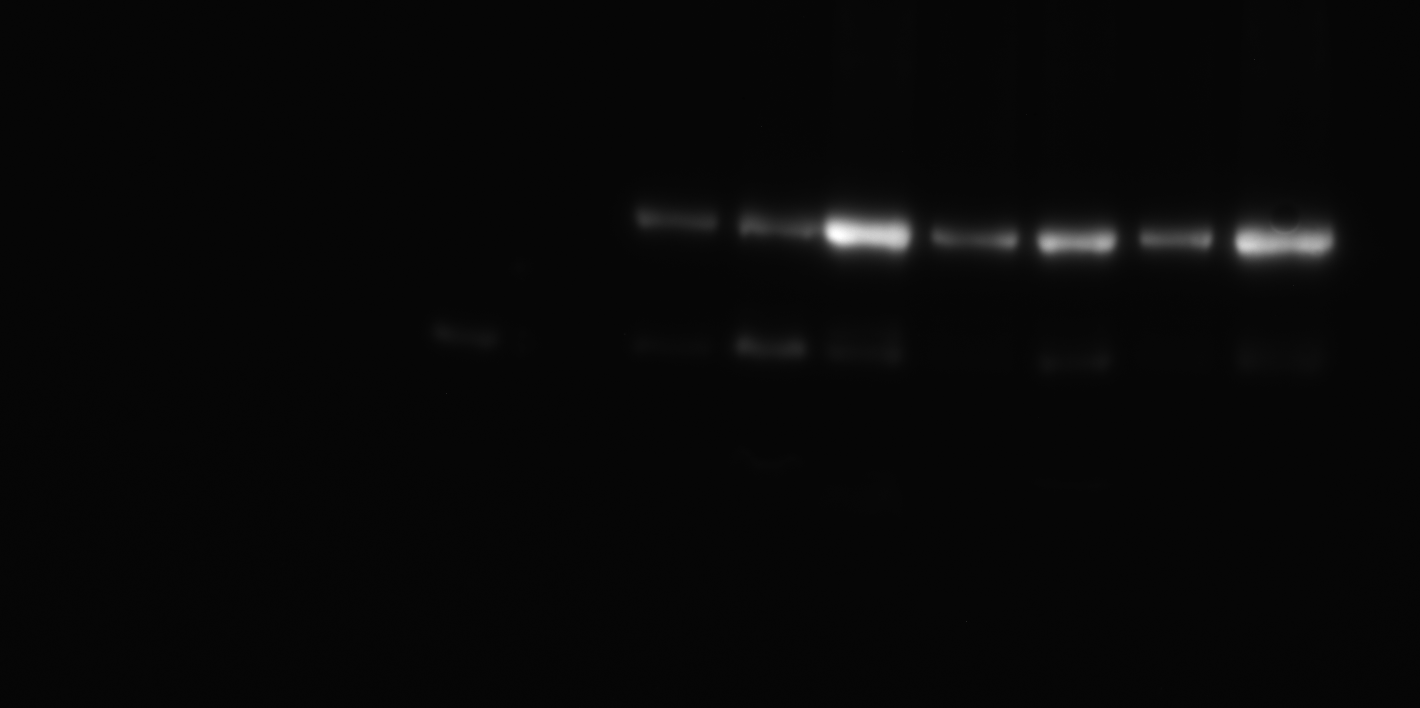

Supplement: Figure 7—figure supplement 2—source data 1. [file elife-60568-fig7-figsupp2-data1.zip › 1_mutations_anti a1,10-10. scan_raw.tif]

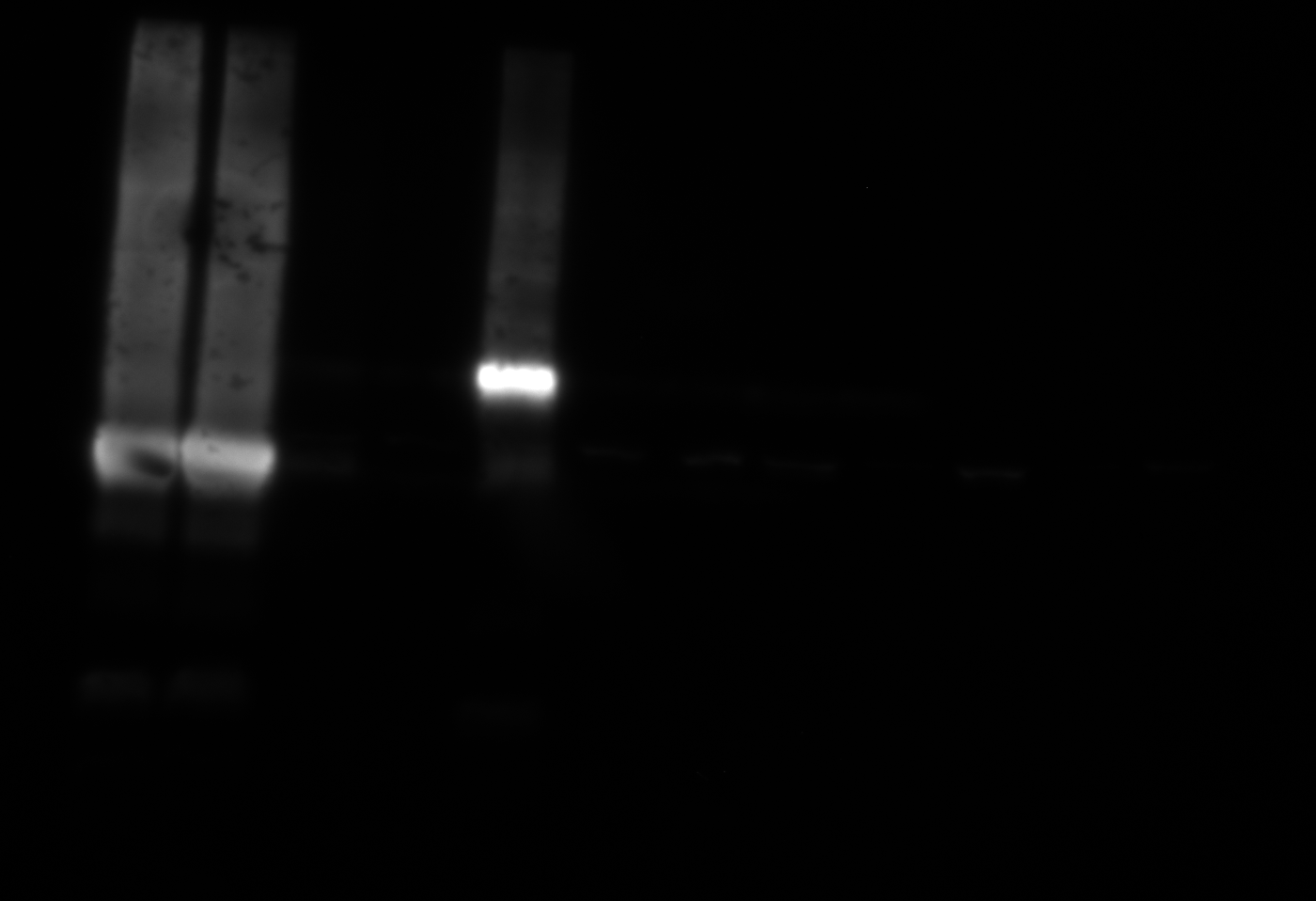

Supplement: Figure 7—figure supplement 2—source data 1. [file elife-60568-fig7-figsupp2-data1.zip › 2_mutations_anti a3_10 secs,3-2. scan_raw.tif]

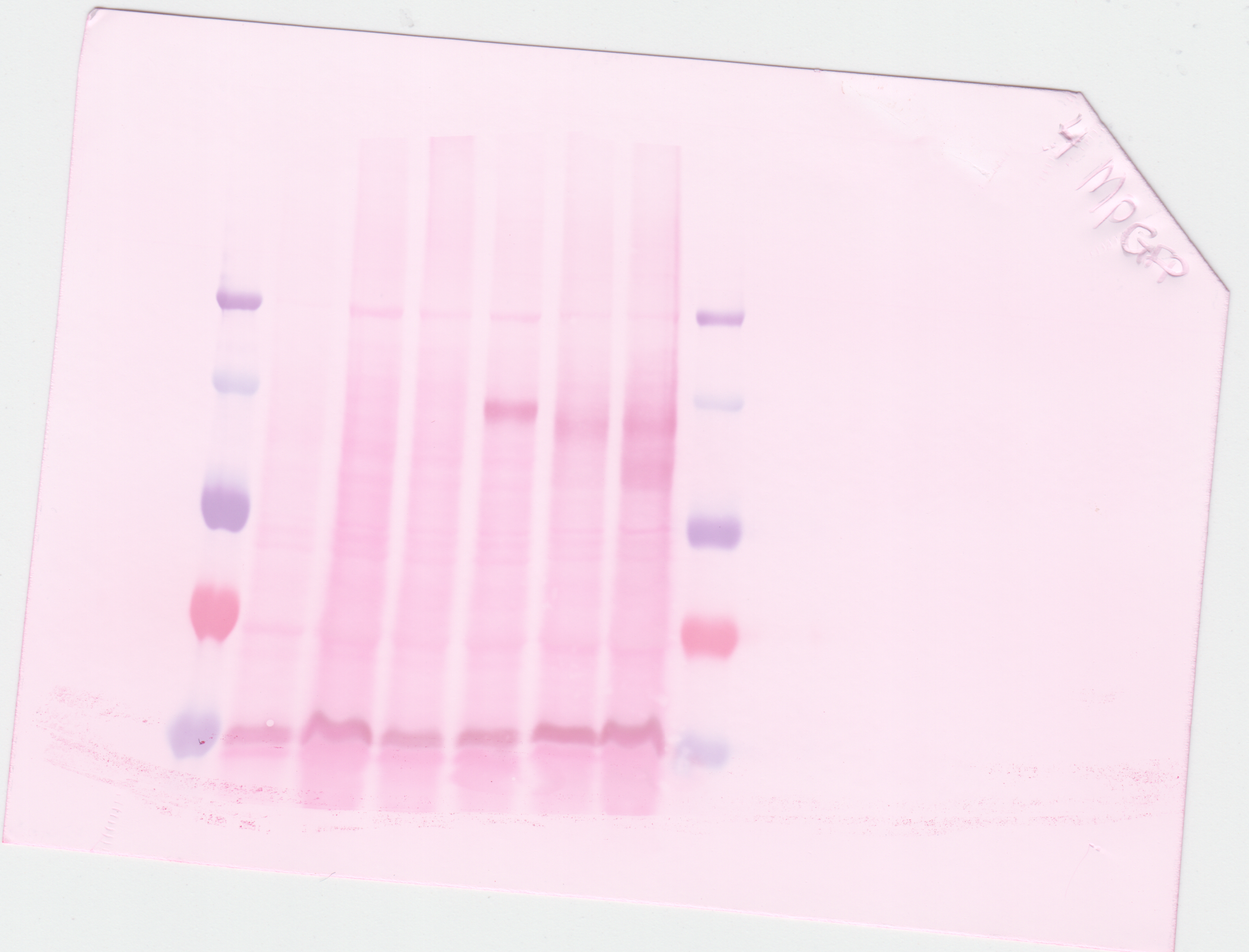

Supplement: Figure 8—source data 2. [file elife-60568-fig8-data2.zip › 4 Mp GFP 001.tif]

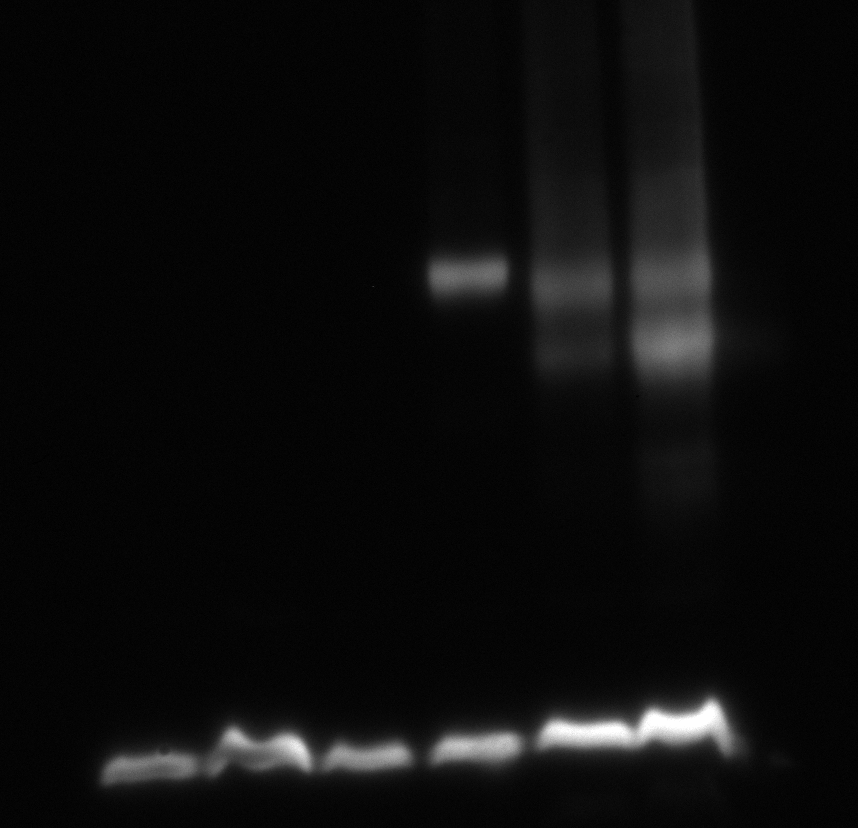

Supplement: Figure 8—source data 2. [file elife-60568-fig8-data2.zip › 4 MpVHA_a_anti VHA_B 300ms,4-3. scan_raw.tif]

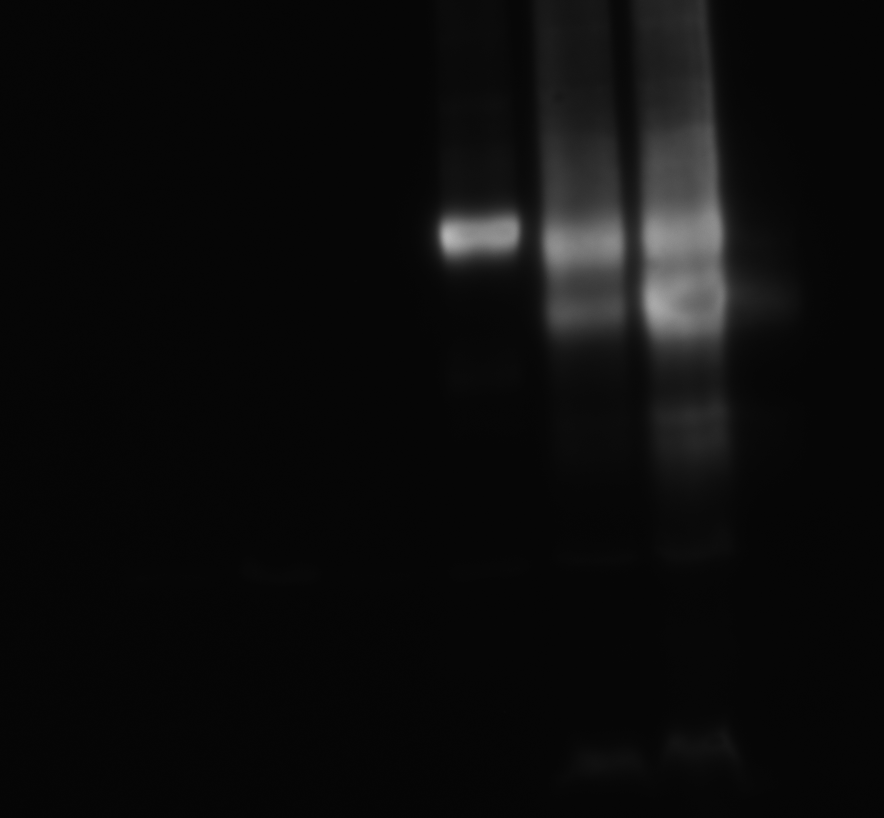

Supplement: Figure 8—source data 2. [file elife-60568-fig8-data2.zip › 4_MpVHA_a anti GFP,11-10. scan_raw.tif]

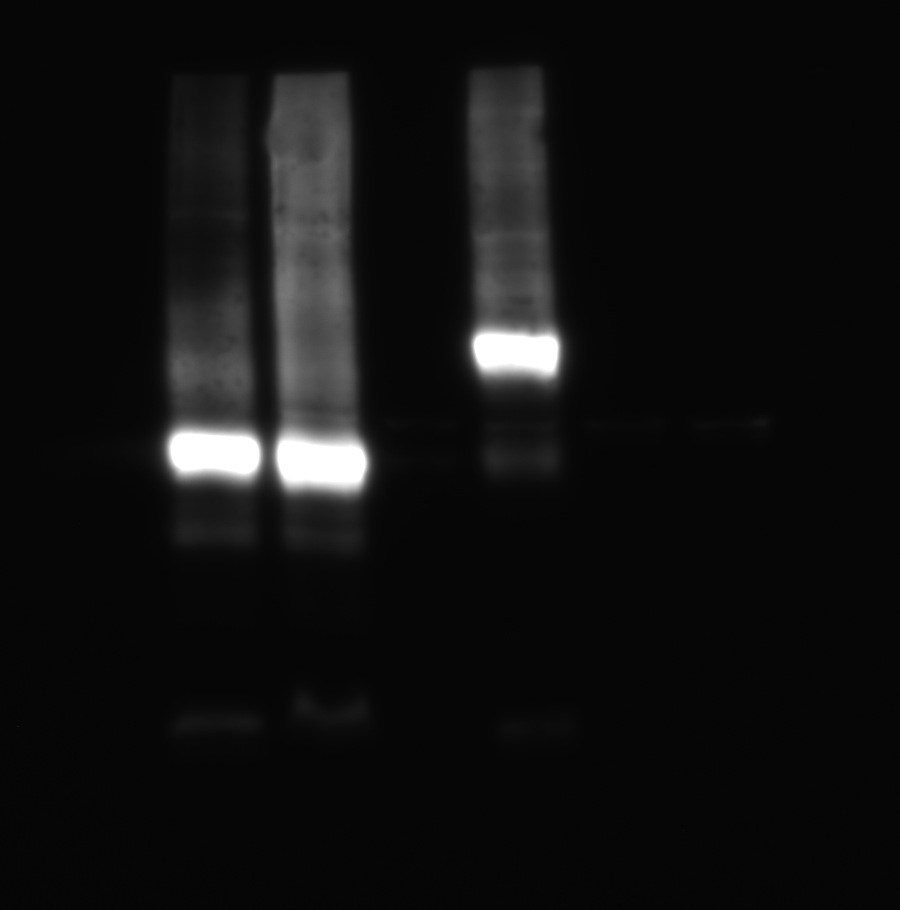

Supplement: Figure 8—source data 2. [file elife-60568-fig8-data2.zip › 5_MpVHA_a anti_a3 10 sec,10-10. scan_raw.tif]
